# Possible Therapeutic Use of Natural Compounds Against COVID-19

**Published:** 2021

**Authors:** Nabab Khan, Xuesong Chen, Jonathan D. Geiger

**Affiliations:** Department of Biomedical Sciences, University of North Dakota School of Medicine and Health Sciences, Grand Forks, North Dakota 58203, USA

**Keywords:** SARS-CoV-2, COVID-19, Endolysosomes, Autophagy, Natural compounds

## Abstract

The outbreak of severe acute respiratory syndrome-coronavirus-2 (SARS-CoV-2) has led to coronavirus disease-19 (COVID-19); a pandemic disease that has resulted in devastating social, economic, morbidity and mortality burdens. SARS-CoV-2 infects cells following receptor-mediated endocytosis and priming by cellular proteases. Following uptake, SARS-CoV-2 replicates in autophagosome-like structures in the cytosol following its escape from endolysosomes. Accordingly, the greater endolysosome pathway including autophagosomes and the mTOR sensor may be targets for therapeutic interventions against SARS-CoV-2 infection and COVID-19 pathogenesis. Naturally existing compounds (phytochemicals) through their actions on endolysosomes and mTOR signaling pathways might provide therapeutic relief against COVID-19. Here, we discuss evidence that some natural compounds through actions on the greater endolysosome system can inhibit SARS-CoV-2 infectivity and thereby might be repurposed for use against COVID-19.

## Introduction

Severe acute respiratory syndrome coronavirus-2 (SARS-CoV-2) is an enveloped virus containing single-stranded RNA genomic material [1,2]. Coronavirus infectious disease-2019 (COVID-19) is a pandemic disease in humans caused by SARS-CoV-2 infection; symptoms and consequences include cardiovascular disorders, acute respiratory distress syndrome (ARDS), and death [3–5]. SARS-CoV-2 infects cells by viral spike proteins interacting with host cells expressing angiotensin-converting enzyme 2 (ACE2) receptors; the virus enters host cells following transmembrane protease serine 2 (TMPRSS2)-mediated priming [6–8]. To infect cells, the virus must be endocytosed into and then released from endolysosomes; a feature common to enveloped viruses [9,10]. In so doing, coronaviruses hijack the endocytic machinery such that they deliver their genomic material at replication sites without initiating host immune detection and host-pathogen responses [8,11–14]. Once released from endolysosomes into the cytosol, coronaviruses replicate in double membrane vesicles that resemble autophagosomes [15–18] and when viral levels are sufficiently high pathological conditions develop including cytokine storms [19–22]. Because endolysosomes are acidic organelles that contain ~60 acid hydrolases capable of catalyzing the degradation of viral particles, enhancing endolysosome acidification might suppress SARS-CoV-2 infection [15,23,24]. The acidic nature of lysosomes regulates the functions of endolysosomes and the autophagy system and multiple endolysosome-associated ion channels and proteins regulate lysosome acidity including vacuolar-ATPase, TRPML1, BK [25], SLC38A9 [26–29], and mammalian target of rapamycin (mTOR) [30–34].

mTOR downstream signaling pathways regulate fundamental cellular processes such as protein synthesis, metabolism, transcription, cell cycle, apoptosis, endolysosomes, autophagy, and immune regulation and tolerance [35–39]. Aberrant mTOR signaling is involved in various pathological conditions such as cancer and inflammation as well as cardiovascular and metabolic disorders [40,41]. In addition, multiple viruses can hijack the mTOR signaling system for the purpose of completing viral replication including influenza [42] and HIV-1 [43, 44] as well as the coronaviruses MERS-CoV [45, 46] and SARS-CoV-2 [15, 47, 48].

The mTOR signaling pathway can be targeted to block the infection and replication of viruses other than coronaviruses by inducing autophagy and inhibiting viral protein synthesis [15,45–47,49,50]. Hence, mTOR might be targeted to suppress SARS-CoV-2 infection and COVID-19 using synthetic and natural compounds [51–57]. Natural compounds (phytochemicals) can enhance endolysosome acidification and autophagy by inhibiting mTOR-signaling pathways [49,58–64]. It has been suggested that increased consumption of phytochemicals or foods rich in phytochemicals might decrease the prevalence and severity of cancer, osteoporosis, and cardiovascular diseases [63]. Fruits, legumes, vegetables, and cereals contain high levels of phytochemicals including carotenoids, terpenoids, phytosterols, flavonoids, isoflavones, isothiocyanates, and fibers; substances shown to have anti-inflammatory, anti-oxidant and anti-infectious properties [64]. Phytochemicals can also enhance the degradative properties of endolysosomes and thereby suppress microbial infections as well as human metabolic and aging-related diseases [15,63,64]. Here, we briefly discuss natural compounds that affect endolysosomes and autophagy, the mTOR sensor, and as such, might find therapeutic use against SARS-CoV-2 infection and the pathogenesis of COVID-19.

## Natural Compounds

### Spermidine and spermine

Polyamines are generated endogenously from arginine and ornithine, and they are ingested as components of various plants [65,66]. Endogenously, putrescine synthesis from ornithine is catalyzed by ornithine decarboxylase [67–69] and from ornithine, the polyamines spermidine and spermine are generated [68]. Exogenously, ingestion of polyamines protected against age-related memory loss [70,71] and rescued memory performance [71,72]. The cardio-protective [73], anti-inflammatory, and antioxidant [74–76], actions of the polyamine spermidine may be mediated by the induction of autophagy [71,77]. Moreover, spermidine and spermine induce 5’-AMP-activated protein kinase (AMPK) and inhibit the mTOR signaling pathway to induce autophagy and suppress functions of inflammatory dendritic cells [78–80]. Spermidine and spermine both inhibited SARS-CoV-2 infection and appeared to do so by inducing viral degradation in endolysosomes [15].

### Resveratrol

Resveratrol is a polyphenol with antioxidant and anti-inflammatory properties, and resveratrol has been found to protect against oxidative damage in high-risk conditions like cancer, diabetes, heart diseases, neurodegenerative diseases, and microbial infections [81]. Resveratrol is enriched in peanuts, berries, and red grapes [81,82], and it can be ingested in capsules containing *Polygonum cuspidatum* plant extracts [83,84]. Resveratrol has an ability to enhance autophagy and kill cancer cells by suppressing the phosphoinositide 3-kinase (PI3K)/A serine/threonine protein kinase (Akt)/mTOR signaling pathway and enhancing AMPK and sirtuin (SIRT1) pathways [85–88]. Resveratrol can exert antiviral effects against various viral infections [89] including herpes simplex virus [90], enterovirus 71, Epstein-Barr virus, respiratory syncytial virus, influenza, and Middle East Respiratory Syndrome-coronavirus (MERS-CoV) [49]; MERS-CoV is a family member of SARS-CoV-2 virus [91,92]. Co-administration of resveratrol with copper may be useful in suppressing SARS-CoV-2 replication and diminishing SARS-CoV-2-induced cytokine storms [93,94].

### Phytoestrogen

Phytoestrogens are natural compounds found in plants such as tofu, flaxseed, soybean, sesame seeds, and garlic [95,96]. Phytoestrogens exert estrogen-like effects [95] and have antioxidant, anti-inflammatory [97–100] and neuroprotective [101,102] properties as well as the ability to induce autophagy [103]. Phytoestrogens restrict PI3K/Akt/mTOR signaling pathways and this mechanism has been implicated in their ability to induce autophagy and kill cancer cells [104–106]. One estrogen, 17β-estradiol, is known already to suppress multiple viral infections including influenza [107], rubella [108], HIV-1 [109], HSV-1 [110], SARS-CoV [111], and SARS-CoV-2 [112–114].

### Trehalose

Trehalose, also known as tremalose and mycose, is a stable disaccharide assembled from two molecules of d-glucose [115]. Some plants, fungi, bacteria, and invertebrate animals can produce trehalose and use it as an energy source as well as to survive freezing and lack of water [116–118]. Trehalose has antioxidant [119] and neuroprotective properties [119–122], and it has been shown to inhibit HIV-1 and *mycobacterium tuberculosis* (Mtb) co-infection by inducing the endolysosomal degradation pathway [123]. Further, trehalose induced mTOR-independent autophagy and suppressed cytomegalovirus infection in different cell types [124].

### Baicalin

Baicalin, a component of *Scutellaria baicalensis* and *Scutellaria lateriflora* [125], can protect against amyloid-β protein-, hydrogen peroxide [H_2_O_2_]-, middle cerebral artery occlusion-, and oxygen/glucose deprivation-induced neurotoxicity [126–131]. At least some of these protective effects might be mediated through its actions on endolysosomes because baicalin can attenuate high-fat diet-induced endolysosome deacidification [132]. Baicalin can also induce apoptosis in cancer cells by downregulating mTOR signaling pathways [133–135]. The anti-influenza [136] effects of baicalin suggests its possible use against SARS-CoV-2 by targeting its 3CL protease enzyme [137].

### Curcumin

Turmeric is a spice with many purported medicinal properties [138] and is a rich source of curcumin [139,140]. Curcumin (1,7-bis (4-hydroxy-3-methoxyphenyl)-1,6-heptadiene-3,5-dione) is also known as diferuloylmethane; a natural polyphenol present in the rhizome of turmeric (*Curcuma longa*) [140,141]. Curcumin has antioxidative and anti-inflammatory properties, and it has been used against arthritis, bacterial infections, metabolic syndrome, anxiety, and hyperlipidemia [142–147]. Curcumin has anti-viral effects against a broad spectrum of viruses including herpes simplex virus-2 (HSV-2) [148], HIV-1, zikavirus [149], influenza virus [149], hepatitis virus [150], and human papillomavirus (HPV) [151]. Moreover, curcumin increases endolysosomal functions by promoting lysosomal acidification and suppressing the mTOR sensor [152–154].

### Quercetin

Quercetin is a flavonoid that is present in many plants and foods including onions, red wine, berries, green tea, apples, ginkgo biloba, and buckwheat [155]. Quercetin has a broad range of biological activities including being anti-inflammatory, attenuating lipid peroxidation, inhibiting platelet aggregation [156–159], inducing cell death in cancer cells by enhancing autophagic flux and lysosomal activity [160], and suppressing PI3K/Akt/mTOR signaling pathways [161–163]. Quercetin displays a broad range of antiviral properties; it interferes with virus entry, replication, and assembly [164–167]. Quercetin can suppress SARS-CoV-2 infection but has yet to be tested against COVID-19 [168].

### Coumarin

Coumarin is a phenolic substance that is a fusion of benzene and α-pyrone rings [169,170]. Coumarin is present in Tonka bean (*D. odorata*) and *Cinnamomum aromaticum* and has also been isolated from various plants [171]. Coumarins have anti-oxidant, anti-bacterial, anti-fungal, anti-viral, and anti-cancer properties [172–175]. A hybrid of phenylsulfonylfuroxan and coumarin induced caspase-dependent cell death, autophagy, and suppressed PI3K/Akt/mTOR signaling pathway to kill cancer cells [176–178]. Accordingly, it has been suggested that coumarin might protect against COVID-19 by blocking the protease enzyme of SARS-CoV-2 [179,180].

### Epigallocatechin 3-gallate (EGCG)

EGCG is a component of tea leaves [181]. EGCG has anti-oxidant properties and may prevent autoimmune diseases and cytokine storms [182–186] by blocking downstream inflammatory signaling pathways of the transcription factors STAT (signal transducer and activator of transcription 1/3) and NF-κB (nuclear factor kappa-light-chain-enhancer of activated B cells) [187–190]. EGCG upregulates AMPK activity in a dose-dependent manner and suppresses mTOR signaling in hepatoma cells [191]. A computer-based study has shown that EGCG is an ATP-competitive inhibitor of Akt/mTOR and enhances autophagy by AMPK activation [192–194]. Moreover, EGCG synergistically enhanced curcumin’s effects on cancer cells by inducing autophagy through suppression of the Akt/mTOR signaling pathway [195].

### Naringenin

Naringenin is a flavorless flavanone; a predominant flavanone in various herbs and fruits including grapefruits, citrus, and tomatoes [196–198]. Naringenin has hepatoprotective, anti-inflammatory, anti-mutagenic, anti-cancer, and anti-microbial [199–204] effects and may control neurological, metabolic, rheumatological, and cardiovascular diseases [205–207]. Moreover, naringenin is an inhibitor of endolysosome two-pore channels (TPCs) [208–210]; channels involved in SARS-CoV-2 and Ebola virus infections [211–213] as well as the ability of HIV-1 protein Tat to escape endolysosomes [214]. Naringenin can induce cancer cell death by promoting autophagy and downregulate the Akt/mTOR signaling pathway [215–219]. These finding suggest a possible use of naringenin against COVID-19 by targeting TPCs and the Akt/mTOR signaling pathway [220–222].

## Conclusion

The COVID-19 pandemic is a global disaster with devasting social, behavioral, economic and health ramifications. Endolysosomes play important roles in regulating SARS-CoV-2 infection and thus might be targeted therapeutically against COVID-19.

Relevant to COVID-19, endolysosomes are important regulators of innate immune responses and antigen presentation and phytochemicals have purported anti-inflammatory, anti-oxidant, and anti-viral properties. These properties might play protective roles in blocking SARS-CoV-2 replication and infection at least in part by enhancing endolysosome acidification, increasing autophagy, and inhibiting mTOR-signaling pathways. Several natural compounds have shown promise in suppressing SARS-CoV-2 infection in humans, but these compounds may be toxic at higher concentrations and doses [223–229]. Accordingly, a great deal more work is necessary to have confidence that phytochemicals can provide therapeutic benefit against SARS-CoV-2 infection and alter positively the clinical course of COVID-19.

## Figures and Tables

**Figure 1:**
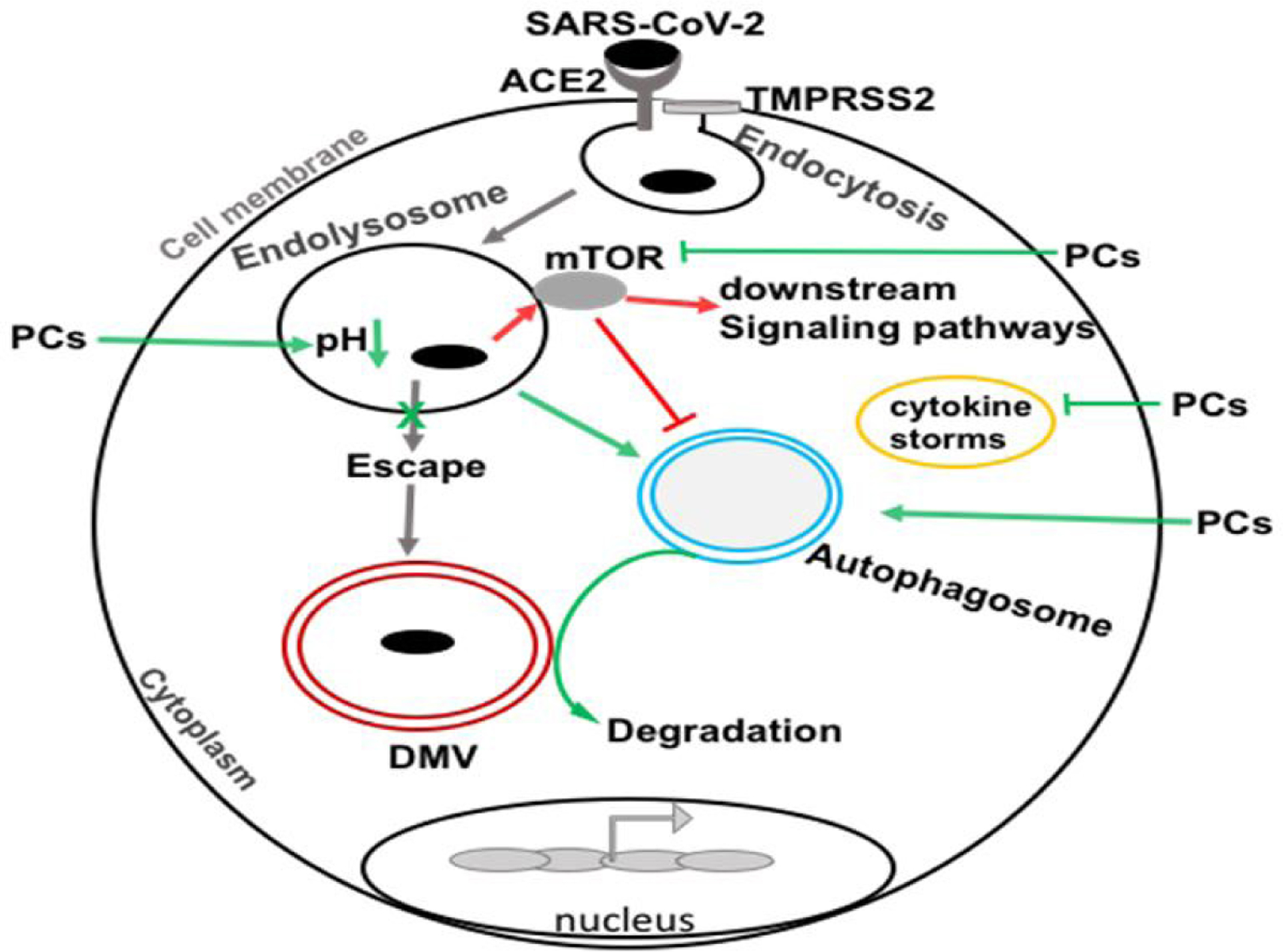
SARS-CoV-2 enters the cell by endocytosis after first interacting with ACE2 and priming by TMPRSS2. During the entry process, the virus escapes from endolysosomes and delivers genomic material at replication sites. The virus replicates in double membrane autophagosome-like vesicles (DMVs) in the cytosol and induces mTOR sensor for exploiting cellular signaling pathways. Natural compounds (phytochemicals; PCs) might suppress SARS-CoV-2 and COVID-19 pathogenesis by augmenting endolysosomes and autophagy degradation pathways through actions on the mTOR sensor, suppressing cytokine storms, and decreasing DMVs formation and viral replication. (Severe acute respiratory syndrome coronavirus-2 (SARS-CoV-2), angiotensin-converting enzyme 2 (ACE2), transmembrane protease, serine 2 (TMPRSS2), double membrane-like vesicles (DMVs), cytokine storm (CS), mammalian target of rapamycin (mTOR).

**Table 1:** Potential natural compounds against SARS-CoV-2 infection and COVID-19 pathogenesis (Scoring according to evidence; +++ (high confidence), ++ (moderate confidence).

Compounds	mTOR inactivation	Endolysosomes and autophagy	Anti-inflammatory	Anti-SARS-CoV-2 activity [References]	Scoring
**Spermidine and spermine**	Negatively regulates mTOR signaling pathway [15,78,79]	Autophagy inducer [15]	Potential anti-inflammatory [74,75]	Restricts SARS-CoV-2 infection by SKP2 modulation (*in vitro*) [15]	++
**Resveratrol**	Negatively regulates mTOR signaling pathways [85–88]	Autophagy inducer [86,230,231]	Potential anti-inflammatory [232]	MERS-CoV inhibition *in vitro* [49]	+++
				SARS-CoV-2 inhibition *in vitro* [93,94]	
				Proposed for clinical trials (NCT04542993)	
**Phytoestrogen**	Negatively regulates mTOR signaling pathways [104–106]	Autophagy inducer [103,104]	Potential anti-inflammatory [97,98,100,233]	Restricts SARS-CoV *in vivo* [111]	+++
				Suggested as a suppressor of COVID-19 [112,114,224,234–236]	
				Estrogen therapy (NCT04539626)	
**Trehalose**	No effect on mTOR [124]	Induces autophagy and lysosomal biogenesis by TFEB activation [120,122]	Potential anti-inflammatory [237]	Potential target against COVID-19 [238]	++
		Induces lysosomes acidification and autophagy by mucolipin-1 (TRPML1) activation to protect mycobacterium tuberculosis infection [123]			
**Baicalin**	Negatively regulates mTOR signaling pathways [133–135]	Autophagy inducer [133]	Potential anti-inflammatory [239–241]	Suppresses COVID-19 pathological condition in vivo, in vitro [137,242–244]	+++
		Induces lysosomes acidification by promoting assembly of v-ATPase pump [132]			
				Proposed for clinical trial (NCT03830684)	
**Curcumin**	Negatively regulates mTOR signaling pathways [152–154]	Autophagy inducer [59,60]	Potential anti-inflammatory [142,143,245]	Proposed against COVID-19 [245–248]	+++
				Proposed for clinical trial against COVID-19 (NCT04353310)	
**Quercetin**	Negatively regulates mTOR signaling pathways [161,162]	Autophagy inducer [160,161]	Potential anti-inflammatory [156,249]	Potential target against COVID-19 [168,250–253]	+++
				Proposed for clinical trial against COVID-19 (NCT04377789)	
**Coumarin**	Negatively regulates mTOR signaling pathways [177]	Autophagy inducer [176,178]	Potential anti-inflammatory [254]	Potential target against COVID-19 (*in silico*) 179,180]	++
**Epigallocat-echin 3-gal-late [EGCG]**	Negatively regulates mTOR signaling pathways [192,194,195]	Autophagy inducer [194,255]	Potential anti-inflammatory [183,185,187,189]	Potential target against COVID-19 and Proposed as previfenon (NCT04446065) [186,256–258]	+++
**Naringenin**	Negatively regulates mTOR signaling pathways [217]	Autophagy inducer [217,219,259]	Potential anti-inflammatory [200,261,262]	Suppresses SARS-CoV-2 infection in vitro [221,222,263]	+++
		A blocker of Two pore channels (TPCs). TPCs are highly involved in SARS-CoV-2’s entry into cells [260]			
